# From one extreme to another- treatment challenges and dilemmas in a transdiagnostic day program

**DOI:** 10.1186/2050-2974-1-S1-O4

**Published:** 2013-11-14

**Authors:** Judith Leahy, Melanie Clark, Cate Borland, Ursula Scott, Rosemary Blundo, Teresa Davids

**Affiliations:** 1 Central Coast Local Health District, NSW Ministry of Health, Australia; 2 Arts for Health, Australia

## 

Can you treat someone with a BMI 15 alongside someone with a BM 48? Will there be more differences than similarities? These were some questions faced when deciding inclusion criteria in our transdiagnositic Central Coast Day Program.

We use Acceptance and Commitment Therapy (ACT) in treatment. The use of a 'values based' approach is helpful in recovery providing 'common ground' for clients who are in the same 'eating disorder boat'.

One aim of treatment is to not restrict or reduce intake of any type of food but rather restore 'freedom around food'.

We use a 'self-observation log', a valuable tool to monitor and analyse emerging patterns between daily food intake, eating behaviours and psychological Room: State.

Our Challenges and dilemmas are:

How do we retain someone with Anorexia Nervosa when cognitively ready for recovery but not 'behaviourally' ready; a Day Program helps contain behaviours but 69% of binges occur in the evening; a client with BED gains an additional 14% body weight in 8 months of treatment; what do you do when someone no longer meets BED criteria but is still overeating and, is it 'better' to just be morbidly obese rather than be morbidly obese and have an eating disorder?

This abstract was presented in the **Adult Treatment and Services** stream of the 2013 ANZAED Conference.

